# Adiponectin in Cerebrospinal Fluid from Patients Affected by Multiple Sclerosis Is Correlated with the Progression and Severity of Disease

**DOI:** 10.1007/s12035-021-02287-z

**Published:** 2021-01-23

**Authors:** Elisabetta Signoriello, Marta Mallardo, Ersilia Nigro, Rita Polito, Sara Casertano, Andrea Di Pietro, Marcella Coletta, Maria Ludovica Monaco, Fabiana Rossi, Giacomo Lus, Aurora Daniele

**Affiliations:** 1 Centro di Sclerosi Multipla, II Clinica Neurologica, Università della Campania “Luigi Vanvitelli”, Via S. Pansini 5, 80131 Naples, Italy; 2 Dipartimento di Scienze e Tecnologie Ambientali Biologiche Farmaceutiche, Università degli Studi della Campania, “Luigi Vanvitelli”, Via G. Vivaldi 42, 81100 Caserta, Italy; 3 CEINGE-Biotecnologie Avanzate Scarl, Via G. Salvatore 486, 80145 Naples, Italy; 4 Dipartimento di Sanità Pubblica, Università degli Studi di Napoli “Federico II”, via Pansini 5, 80145 Naples, Italy

**Keywords:** Adiponectin, HMW oligomers, Multiple sclerosis, Cerebrospinal fluid, Inflammation

## Abstract

Adiponectin exerts relevant actions in immunity and is modulated in several disorders, such as multiple sclerosis (MS). In this study, we characterized adiponectin expression and profiles in cerebrospinal fluid (CSF) from MS patients to investigate its potential relationship with the severity and progression of the disease. Total adiponectin in CSF was measured by ELISA in 66 unrelated CSF MS patients and compared with 24 age- and sex-matched controls. Adiponectin oligomer profiles were analysed by Western blotting and FPLC chromatography. Total CSF adiponectin was significantly increased in MS patients compared with controls (9.91 ng/mL vs 6.02 ng/mL) (*p* < 0.001). Interestingly, CSF adiponectin positively correlated with CSF IgG, and CSF/serum albumin directly correlated with CSF/serum adiponectin. Our data demonstrated that CSF adiponectin predicts a worse prognosis: patients with the progressive form of MS had higher levels compared with the relapsing remitting form; patients with higher EDSS at baseline and a higher MS severity score at 4.5-year follow-up had significantly elevated adiponectin levels with respect to patients with a less severe phenotype. Finally, the adiponectin oligomerization profile was altered in CSF from MS patients, with a significant increase in HMW and MMW. The correlation of CSF adiponectin with the severity and prognosis of MS disease confirmed the role of this adipokine in the inflammatory/immune processes of MS and suggested its use as a complementary tool to assess the severity, progression and prognosis of the disease. Further studies on larger MS cohorts are needed to clarify the contribution of adiponectin to the etiopathogenesis of MS.

## Background

Multiple sclerosis (MS) is an autoimmune disease of the human central nervous system (CNS) that is very often accompanied by unpredictable clinical relapses and remissions and/or by disability progression over time. The aetiology remains unclear, but the main MS pathological features are due to inflammatory attacks that lead to neurodegenerative processes [1, 2]. Cerebrospinal fluid (CSF) protects the central nervous system (CNS) in different ways: regulation of metabolic homeostasis, supply of nutrients, adjuvant of the lymphatic system and regulation of intracranial pressure [3]. In addition, CSF represents the “gold standard” laboratory sample for the diagnosis of MS through the presence of oligoclonal bands, a symptom of CNS inflammation [4, 5]. Indeed, the analysis of CSF markers permits the evaluation of inflammatory processes in MS [6].

The main trademarks of MS are triggered by the interaction between genetic and environmental risk factors. In particular, it is well known that overweight and obesity are risk factors for MS, and an increase in their prevalence has been reported among patients with MS [7]. In this context, adipose tissue, through dysregulated secretion of adipokines, plays a pivotal role in the development and establishment of several inflammation-related processes [8, 9]. Adipokines have been proposed as the molecular link between adipose tissue and other organs/tissues involved in inflammatory-immunologic activation, including the CNS [10–12]. Among the adipokines, adiponectin is a relevant serum adipokine with protective effects against a variety of pathophysiological conditions, especially metabolic diseases [13, 14]. In healthy subjects, adiponectin levels are approximately 5–30 μg/mL, representing 0.01% of the total serum proteins. Adiponectin circulates as oligomers of different weights: low (LMW), medium (MMW) and high molecular weight (HMW) [14, 15]. The complex biological structure of adiponectin determines the distinct functional effects of the different oligomers, with the HMW adiponectin eliciting the most potent biological effect [16]. Adiponectin expression is decreased in metabolic disorders, such as obesity and related diseases [17]; in contrast, in autoimmune diseases, such as MS and rheumatoid arthritis, its expression is increased and correlated with a more severe prognosis [18, 19].

Until now, few data have been reported about adiponectin expression in CSF either in normal subjects or in MS subjects [20–24].

The aim of the present study was to characterize adiponectin expression levels and its oligomerization profile in CSF from MS patients compared with those of normal controls. Moreover, the relationship between CSF adiponectin levels and clinical-biochemical MS phenotypes was analysed to investigate the association of this adipokine with the activity and severity of the disease.

## Methods

### Patients

Sixty-six MS patients (38 females, 28 males) were recruited at the moment of diagnosis at the Multiple Sclerosis Center, Second Division of Neurology of University of Campania “Luigi Vanvitelli” and observed prospectively over time (4.5 years).

The inclusion criteria were MS diagnosis according to the revised McDonald criteria [25], age ≥ 18 and ≤ 65 years and treatment-free period of 1 month with immunoglobulins and/or steroids. The exclusion criteria were chronic disease of the immune system other than MS; presence of metabolic diseases; active systemic bacterial, viral or fungal infections; previous disease-modifying treatment (DMT); and pregnancy or nursing (lactating) women. Standard lumbar puncture was performed for each patient at the same time in the middle of the morning after fasting for 12 h by an experienced neurologist; cerebrospinal fluid (CSF) (~ 4–5 mL) was collected.

As controls, 24 subjects (17 males, 11 females) and age-, body weight- and body mass index (BMI)-matched patients who underwent lumbar puncture for other suspected diseases (but with no confirmation of MS or other autoimmune or infectious diseases at the end of the diagnostic protocol) were recruited. Healthy controls followed the same inclusion and exclusion criteria. CSF was collected from patients at the moment of MS diagnosis before starting any treatment. According to clinical practice, demographic and clinical characteristics and body mass index (BMI) of all participants were recorded; every patient underwent extensive clinical and disability evaluation with the Expanded Disability Status Scale (EDSS), brain and spinal cord magnetic resonance imaging (MRI), lumbar puncture with oligoclonal band evaluation, extensive autoimmune panel and metabolic evaluation [26]. The total annualized relapse rate (ARR) was calculated as the total number of clinical relapses that occurred the last year before the diagnosis. At baseline, the patients were considered active if at the moment of sampling they had any relapses and any activity on MRI (presence of contrast enhancement). The EDSS was used to assess disability at baseline and every 6 months. The progression index (PI) and multiple sclerosis severity score (MSSS) were used to calculate MS severity. PI was defined as the current EDSS score divided by disease duration (years from onset to last clinical evaluation). MSSS is an algorithm that relates EDSS scores to the distribution of disability in patients with similar disease durations [27].

### Anthropometric and Adiponectin Measurements

For each subject, height and weight were obtained using standard techniques; body mass index (BMI) was calculated as body weight (kg)/height^2^ (m^2^). Adiponectin concentrations were measured in CSF by enzyme-linked immunosorbent assay (ELISA) (Biovendor R&D, USA). Each CSF sample, diluted 1:10, was assayed three times in duplicate. Serum adiponectin data were obtained in a previous study [18].

### Western Blot Analysis

Five microlitres of CSF was treated with 1× Laemmli buffer, heated at 95 °C for 2 min, loaded on a 10% SDS-PAGE gel and transferred as previously described [28]. The blots were developed by ECL (Amersham Biosciences, NJ, USA) with the use of Kodak BioMax Light film, digitalized with a scanner (1200 dpi) and analysed by densitometry with ImageJ software (http://rsbweb.nih.gov.ij/). Each sample was tested three times in duplicate.

### Gel Filtration Analysis

The oligomeric distribution of adiponectin in CSF samples was analysed by gel filtration chromatography (FPLC) on a Superdex 200 10/300 GL column connected to a Fast Protein Liquid Chromatography system (Amersham Pharmacia Biotech, Uppsala, Sweden). In detail, 500 μL CSF was eluted at 0.5 mL/min using PBS 100 mmol/L, pH 7.4. Fractions (500 μL) were collected, and the occurrence of adiponectin oligomers in each fraction was tested using both ELISA and Western blotting analysis. The column was calibrated using ferritin (440 kDa), aldolase (158 kDa) and ovalbumin (44 kDa) (GE Healthcare).

### Statistical Analysis

Continuous variables are given as the mean and standard deviation, and categorical variables are given as absolute and relative frequencies. Univariate analysis was performed using parametric (Student’s *t* test for independent samples) and nonparametric statistics (Mann-Whitney *U* test) for continuous variables. Fisher’s exact test was performed for categorical variables. The potential association between higher and low levels of adiponectin in determining clinical outcome in MS patients was assessed using a univariate model. Odds ratios (ORs), with 95% confidence intervals (CIs), were estimated by a logistic regression model with the significant variables of the univariate model as covariates. Spearman correlation was used to investigate the relationship between CSF adiponectin and other inflammatory CSF markers. A *p* value of < 0.05 was considered statistically significant.

## Results

### Anthropometric and Clinical Features

The anthropometric and demographic characteristics of MS patients at baseline and of healthy controls are illustrated in Table 1. No statistically significant differences in anthropometric parameters were found between patients and controls.

**Table 1:** Anthropometric and demographic characteristics of MS patients compared with controls

	MS patients *n* = 66	Controls *n* = 24	*P* value
Age	43.29 ± 16.15	37.27 ± 12.64	0.60
Sex (M/F)	28/38	11/17	0.80
BMI	25.45 ± 5.88	25.30 ± 4.98	0.53

Table 2 shows the clinical and instrumental characteristics of MS patients at the moment of diagnosis. In summary, the majority of patients (84.8%) had relapsing remitting multiple sclerosis (RRMS), and ten patients had the progressive form of the disease.

**Table 2:** Clinical and biochemical characteristics of MS patients

Clinical and biological variables	MS patients *n* = 66
ARR previous year (mean ± ds)	1.18 ± 2.01
PI (mean ± ds)	2.02 ± 4.62
Basal EDSS (mean ± ds)	1.97 ± 1.6
MSSS (mean ± ds)	3.49 ± 2.81
Disease duration (mean ± ds)	2.98 ± 3.04
EDSS at the end of follow-up (mean ± ds)	1.80 ± 1.71
Follow-up (years) (mean ± ds)	4.55 ± 1.49
Relapsing remitting MS (RR)	84.8%
CSF IgG (mg/dL)	5.59 ± 7.50
Oligoclonal bands (mean)	0.55 ± 0.49
Serum IgG (mg/dL)	1067.48 ± 316.47
Serum adiponectin (μg/mL)^a^	11.86 ± 3.53
CSF adiponectin/serum adiponectin	0.85 ± 0.64
Serum albumin μg/dL	4.42 ± 0.57
Link Index (CSF IgG/serum IgG × serum albumin/CSF albumin)	0.80 ± 0.48
Barrier Index (CSF albumin/serum albumin × 100)	10.98 ± 6.09

^a^Data obtained in a previous study [ref. 18]

### Total Adiponectin Concentration and Association with Clinical Parameters

**Fig. 1:**
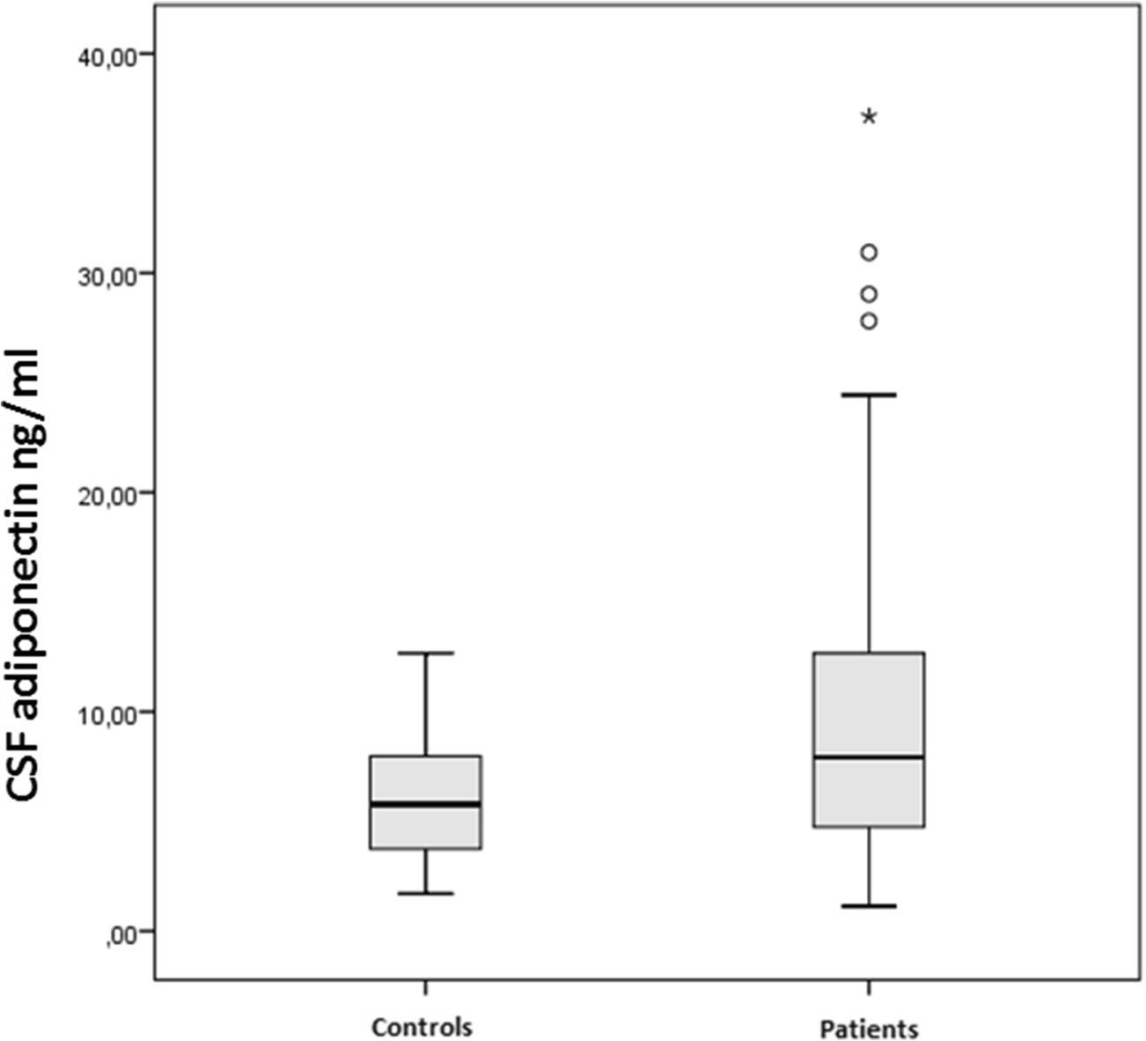
ELISA showed that total CSF adiponectin levels were significantly higher in MS patients than in controls (9.91 ± 7.53 vs 6.02 ± 2.74, *p* value = 0.009)

As shown in Fig. 1, total CSF adiponectin levels were significantly higher in MS patients than in controls (9.91 ± 7.53 vs 6.02 ± 2.74, *p* value = 0.009). Subsequently, we investigated the relationship between CSF adiponectin and the clinical phenotype/severity of the disease: we found significantly higher CSF adiponectin levels in progressive patients than in relapsing patients (15.51 ± 11.42 vs 8.91 ± 6.25 *p* = 0.01), with no differences for sex (female/male) or active/inactive patients.

Taking into account that the CSF albumin/serum albumin ratio (barrier index) is a marker of blood–CSF barrier integrity [29], MS patients were divided into two subgroups according to the barrier index (above or below the 0.45 normal cut-off). Compared with those without CSF barrier damage, patients with CSF barrier damage had higher CSF adiponectin, although the difference was not significant (11.28 ± 9.25 vs 8.63 ± 5.28) (*p* = 0.67).

In addition, the median value of CSF adiponectin (9.91 μg/mL) was used as an arbitrary cut-off to divide patients at the end of the follow-up period into two subgroups. Using the univariate model and comparing the two MS patient subgroups, we found that patients with CSF adiponectin levels above the arbitrary cut-off had significantly higher BMI, serum adiponectin, MSSS and EDSS at the end of the 4.5-year follow-up (see Table 3). Interestingly, we found that the CSF adiponectin/serum adiponectin ratio directly correlated with the CSF albumin/serum albumin ratio (*p* = 0.04).

**Table 3:** Univariate analysis of clinical characteristics of MS patients according to high or low levels of CSF adiponectin; the median value of adiponectin (9.91 μg/mL) was used as an arbitrary cut-off)

	High CSF adiponectin (ng/mL)	Low CSF adiponectin (ng/mL)	*p* value
Age	40.72 ± 14.87	35.17 ± 10.73	0.71
Sex (% female/male)	37.8%/42.8%	62.2%/57.14%	0.47
BMI	27.22 ± 5.15	24.49 ± 4.75	0.04
Serum adiponectin (μg/mL)^a^	12.75 ± 2.17	11.32 ± 4.08	0.01
MSSS	4.23 ± 3.05	2.41 ± 2.16	0.04
Progression Index	2.21 ± 4.45	1.84 ± 4.83	0.40
EDSS at baseline	2.27 ± 1.88	1.71 ± 1.29	0.35
EDSS at the end of follow up	2.35 ± 1.98	1.33 ± 1.30	0.03

^a^Data obtained in a previous study [ref. 18]

In addition, the multivariate analysis (see Table 4) indicated that patients with higher levels of CSF adiponectin had a higher risk of MSSS disease progression (OR 1.72 95%, IC 1.05–2.79, *p* = 0.02).

**Table 4:** Multivariate analysis confirms that patients with higher CSF adiponectin levels have a higher risk of disability progression (MSSS), independently of body mass index (BMI)

	OR (IC 90%)	*p* value
Age	1.03 (0.97–1.09)	0.22
BMI	0.91 (0.78–1.05)	0.20
Serum adiponectin (μg/mL)	1.41 (0.97–2.07)	0.07
MSSS	1.72 (1.05–2.79)	0.02
EDSS at the end of follow-up	0.62 (0.27–1.39)	0.25

**Fig. 2:**
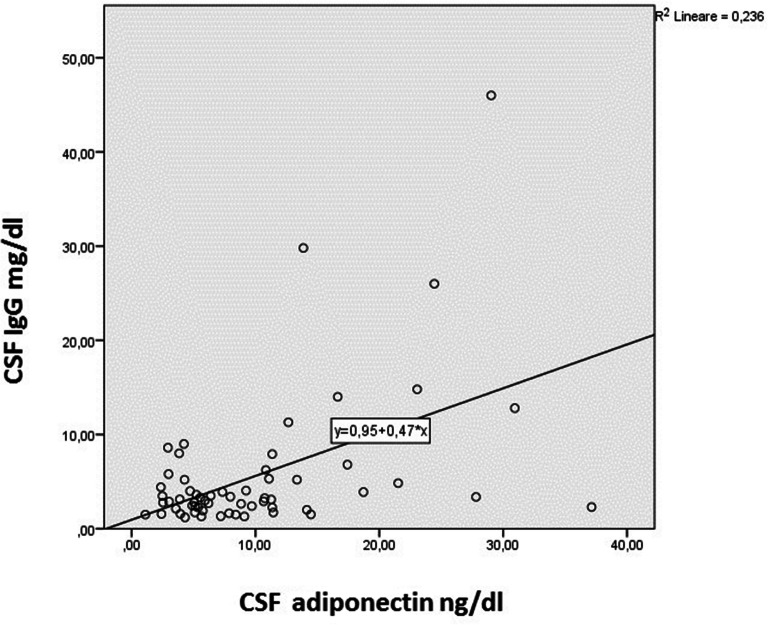
CSF adiponectin directly correlates with CSF IgG

Finally, we investigated the relationship between CSF adiponectin and other inflammatory parameters, such as CSF IgG, CSF albumin, Link Index and oligoclonal bands, and found that CSF adiponectin was strongly correlated with CSF IgG (*p* = 0.02) but not with other parameters (see Fig. 2).

### Oligomeric Distribution of Adiponectin in CSF

**Fig. 3:**
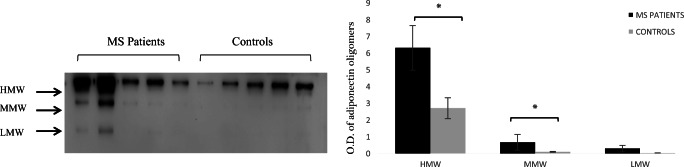
Adiponectin high molecular weight (HMW) and medium molecular weight (MMW) oligomers are significantly higher in patients with multiple sclerosis (MS) than in controls. **a** Representative Western blot for total adiponectin and its different oligomers [HMW, MMW, low molecular weight (LMW)] in the serum of five controls and five patients with MS. **b** Pixel quantization of all controls and all patients with MS. Values are reported as percentages compared with the control. *P < 0.05

Adiponectin oligomer distributions were analysed by both Western blotting and FPLC analysis. The first analysis showed three bands corresponding to HMW (≥ 250 kDa), MMW (≥ 180 kDa) and LMW (≥ 70 kDa) in CSF from both controls and MS patients (Fig. 3). In addition, adiponectin and its oligomers were significantly increased in MS patients (Fig. 3a), as indicated by the densitometric analysis (Fig. 2b; *p* < 0.05).

**Fig. 4:**
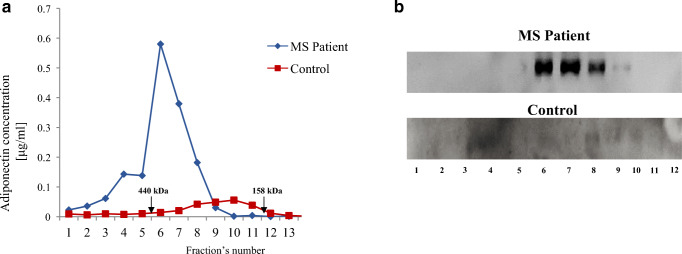
Adiponectin oligomers are increased in CSF from MS patients compared with normal controls. Adiponectin oligomers in each fraction obtained from fast protein liquid chromatography analysis and subjected to ELISA (**a**) and Western blotting (**b**)

Successively, the adiponectin oligomeric profile was further characterized by FPLC under native conditions. Both ELISA (Fig. 4a) and Western blotting (Fig. 4b), performed on each FPLC fraction, confirmed that adiponectin levels are higher in MS patients than in controls. Notably, HMW and MMW, the oligomers that elicit the more potent biological effects, were predominantly increased.

## Discussion

In this study, we analysed the expression of adiponectin and characterized its oligomeric profile in CSF from MS patients. We found that CSF levels of adiponectin and its oligomers were increased in MS patients compared with control subjects matched for age, sex and BMI. In addition, we found that CSF adiponectin is correlated with the severity and progression of the disease and that CSF adiponectin levels are positively associated with MSSS, EDSS and CSF IgG (*p* = 0.02); furthermore, the CSF adiponectin/serum adiponectin ratio correlates with the CSF albumin/serum albumin ratio, a measure of the integrity of the blood–brain barrier. Altogether, our results support the pivotal role of adiponectin in the inflammatory/immune processes of MS.

The analysis of CSF offers the opportunity to directly evaluate the specific inflammatory processes of MS through the dosage of different cytokines [5]. Indeed, the establishment of cellular and myelin damage, the main pathological trademarks of MS, is due to the production of a large amount of proinflammatory cytokines by CD4+ T cells [30]. In this context, adipose tissue reacts and adapts itself through the release of adipokines that act under different physio-pathological conditions, such as the regulation of energy balance, inflammatory state and immune response [8, 31, 32].

Previously, it has been demonstrated that serum adiponectin levels are increased in MS patients compared with healthy controls and are significantly correlated with higher activity of the disease (total ARR) and worse prognosis (PI and MSSS) [18]. The presence of adiponectin in CSF has been previously analysed with contrasting results regarding the expression of the different oligomers; to summarize, in healthy subjects, only the medium- and low-molecular-weight complexes seem to be found in CSF [20, 24]. Furthermore, CSF adiponectin levels are positively correlated with systemic serum levels, with the CSF/serum adiponectin ratio correlated with the CSF/serum albumin ratio, indicating that CSF adiponectin might represent an additional marker of blood–brain barrier damage in MS patients [20, 33].

Adiponectin expression in CSF has also been studied in other neurodegenerative diseases, such as Alzheimer’s disease and mild cognitive impairment; it has been found to be positively correlated with disease markers such as Aβ42 and cognitive function [34, 35]. Regarding MS, to our knowledge, there are only two published studies reporting adiponectin expression in CSF. In accordance with our results, Neumeier et al. found that CSF adiponectin directly correlates with serum concentrations and that the CSF/serum adiponectin ratio correlates to the CSF/serum albumin ratio [33]. Similarly, Hietaharju et al. reported that adiponectin levels in the CSF of MS patients are significantly higher than those in control subjects [36]. The data presented in this work, together with published data, strongly suggest that CSF and serum adiponectin might be considered markers of MS severity. However, although IgG levels and the Ig index are still valid parameters, CSF K free light chains and K index have higher accuracy and predictive value with respect to MS disability progression. Therefore, future validation studies will be needed to analyse the possible association between adiponectin and K indexes [37, 38].

To our knowledge, this is the first study analysing the implication of adiponectin oligomers in CSF from MS patients. Our data show a major involvement of HMW and MMW oligomers, which are increased in patients compared with controls. Previously published papers described the presence of adiponectin in CSF with contrasting results regarding the expression of different oligomers; medium- and low-molecular-weight complexes seem to be found in CSF. It is important to emphasize that adiponectin circulates as a full-length version and as a globular domain, which has shown very high biological activity [39]. In addition, it has been demonstrated that the globular domain of adiponectin successfully attenuates the MS murine phenotype and is considered an effective therapeutic tool [40]. Here, we could not find the globular form of adiponectin in CSF from either patients or controls, probably due to a limitation of the laboratory technique. On the other hand, to our knowledge, there are no literature data describing globular adiponectin.

However, the biological role of adiponectin in MS is still a matter of debate. In vitro studies demonstrated that adiponectin biologically functions in the brain in processes related to MS, such as synaptic activity and regulation of the immune response [41]. Regarding the regulation of the immune response, it has been proposed that adiponectin can inhibit Th17 cell-mediated autoimmune CNS inflammation, which typically occurs in MS [42]. Zhang et al. confirmed this hypothesis, demonstrating that adiponectin deficiency promotes CNS inflammation and demyelination with the development of exacerbated autoimmune encephalomyelitis (EAE), an animal model of human MS [42]. Strengthening this hypothesis, Piccio et al. showed that adiponectin deficiency leads to a worsening of the MS murine phenotype [40].

## Conclusions

In conclusion, we demonstrated that the baseline levels of adiponectin in CSF from MS patients are correlated with the severity and progression of disease. This finding, together with the strong correlation of CSF adiponectin with CSF IgG, confirms the pivotal role of adiponectin in the inflammation/immune responses of MS. Further studies are required to clarify the contribution of adiponectin to the complex cytokine network working in MS, but altogether, our data outline the importance of this adipokine in the etiopathology as well as in the outcome of MS disease.

Adding new knowledge to larger MS cohorts could reinforce adiponectin as a tool to assess the severity, progression and prognosis of this disorder.

## Data Availability

Not applicable.
