# Correction to: Adiponectin in Cerebrospinal Fluid from Patients Affected by Multiple Sclerosis Is Correlated with the Progression and Severity of Disease

**DOI:** 10.1007/s12035-021-02331-y

**Published:** 2021-02-18

**Authors:** Elisabetta Signoriello, Marta Mallardo, Ersilia Nigro, Rita Polito, Sara Casertano, Andrea Di Pietro, Marcella Coletta, Maria Ludovica Monaco, Fabiana Rossi, Giacomo Lus, Aurora Daniele

**Affiliations:** 1 Centro di Sclerosi Multipla, II Clinica Neurologica, Università della Campania “Luigi Vanvitelli”, Via S. Pansini 5, 80131 Naples, Italy; 2 Dipartimento di Scienze e Tecnologie Ambientali Biologiche Farmaceutiche, Università degli Studi della Campania, “Luigi Vanvitelli”, Via G. Vivaldi 42, 81100 Caserta, Italy; 3 CEINGE-Biotecnologie Avanzate Scarl, Via G. Salvatore 486, 80145 Naples, Italy; 4 Dipartimento di Sanità Pubblica, Università degli Studi di Napoli “Federico II”, via Pansini 5, 80145 Naples, Italy

**Correction to: Mol Neurobiol**

10.1007/s12035-021-02287-z

The original version of this article unfortunately contained some mistakes.

The surnames and given names of authors were interchanged. It should be:

Elisabetta Signoriello, Marta Mallardo, Ersilia Nigro, Rita Polito, Sara Casertano, Andrea Di Pietro, Marcella Coletta, Maria Ludovica Monaco, Fabiana Rossi, Giacomo Lus, and Aurora Daniele

The original article has been corrected.

